# Prevalence and Risk Factors of Internet Gaming Disorder Among Female Secondary School Students in Al-Ahsa, Kingdom of Saudi Arabia

**DOI:** 10.7759/cureus.40375

**Published:** 2023-06-13

**Authors:** Hanin S Bumozah, Abdulkareem J Al-Quwaidhi, Rahmah AL-Ghadeeb

**Affiliations:** 1 Preventive Medicine, Al-Ahsa Health Cluster, Ministry of Health, Al-Ahsa, SAU

**Keywords:** female adolescents, anxiety, depression, secondary school, prevalence, saudi arabia, al-ahsa, risk factors, video games, internet gaming disorder

## Abstract

Objectives: This study measured the prevalence and potential risk factors of Internet gaming disorder (IGD) among female secondary school students in Al-Ahsa, Kingdom of Saudi Arabia.

Methods: This cross-sectional study was conducted between January and February 2023. A total of 400 female secondary school students in Al-Ahsa were recruited through multistage stratified cluster sampling. Data were collected by distributing a self-administered questionnaire among students. A chi-squared test was performed to compare categorical variables. The associations between IGD, depression, and anxiety scores were determined using the Pearson correlation coefficient.

Results: Among the total sample, 282 were classified as "gamers" and included in our analysis. The prevalence of IGD was found to be 19%. We also found a statistically significant and moderately positive correlation between IGD and anxiety and depression scores. Spending more than four hours daily playing video games, starting to play video games at less than seven years of age, having depression, having anxiety, and playing an online game were found to be potential risk factors for IGD.

Conclusion: IGD among female adolescent students in Al-Ahsa is a public health concern that requires attention from the concerned parties. Health education projects on IGD, its risk factors, and its consequences should be designed for adolescents and their families.

## Introduction

The number of internet users has rapidly increased worldwide in the last few years. This number reached 5.2 billion, increasing by 1.9% from 2022 to 2023 [1]. In Saudi Arabia, the number of Internet users was estimated at 36.3 million at the beginning of 2023, which is approximately 500,000 more users than those in the previous year [1]. Excessive use of the Internet and electronic devices significantly affects public health [2,3].

One aspect of Internet usage is online gaming and game addiction is widely discussed among researchers and healthcare professionals [4,5]. Globally, the problem has become increasingly apparent, involving a significant portion of children and adolescents, and it varies in different countries and among specific populations, with some estimates reaching up to 50% or even more [6]. A recent systematic review and meta-analysis of 53 studies conducted in different countries showed that the pooled prevalence of Internet gaming disorder (IGD) is 3.05% [7]. Published in 2020, another systematic review of 160 studies reported a prevalence of 0.21%-57.5% [8]. The prevalence of IGD varies between countries and studies because of differences in assessment tools, study populations, and diagnostic criteria [9].

In 2018, gaming disorder was included as a clinically significant syndrome in the eleventh edition of the International Classification of Diseases (ICD-11) [2]. Recently, the ICD-11 stated that Internet gaming disorder (IGD) is diagnosed "when the pattern of gaming behavior is of such a nature and intensity that it results in marked distress or significant impairment in personal, family, social, educational, or occupational functioning" [2,10]. Published by the American Psychiatric Association (APA) in 2013, the Diagnostic and Statistical Manual of Mental Disorders (DSM-5) defines IGD as "the persistent and recurrent use of the Internet to engage in games, often with other players, leading to clinically significant impairment or distress" [6,11]. The APA lists these as the symptoms of IGD: being preoccupied with gaming, having withdrawal symptoms, having tolerance, being unable to quit, continuing to game despite problems, experiencing family problems due to prolonged gaming, gaming to relieve negative moods, and jeopardizing or losing a job or relationship due to gaming [4]. If an individual experiences five or more of these symptoms within a year, they can be diagnosed with IGD [4]. Studies have shown that IGD affects male children five times more than it affects female children [9], and the most affected are individuals aged between 12 and 20 [9]. IGD brings several negative outcomes that can range from psychological and mental health problems to academic and somatic health complications, such as pain in the wrist, elbow, and neck, and being overweight or obese [9]. Furthermore, it has been found that heavy gamers (those who play for more than five hours daily) are at a higher risk of depression and suicidal ideation [9].

Several tools that cover the criteria of both the DSM-5 and ICD-11 have been used to measure IGD [10]. Some of these tools are the Petry IGD, the Internet Gaming Disorder Scale-Short Form (IGDS9-SF), the Personal Internet Gaming Disorder Evaluation-9 (PIE-9), the Ten-Item Internet Gaming Disorder Test (IGDT-10), the Structured Clinical Interview for Internet Gaming Disorder (SCI-IGD), the Clinical Video Game Addiction Test 2.0 (CVAT2.0), the Internet Gaming Use-Elicited Symptom Screen (IGUESS), and the Diagnostic Interview for Internet Addiction (DIA). Tools with greater evidential support are the Seven-item Gaming Addiction Scale (GAS-7), IGDS9-SF, IGDT-10, Young’s Diagnostic Questionnaire (YDQ), and Lemmens Internet Gaming Disorder Scale-9 [10]. In Saudi Arabia, the prevalence of IGD has been reported by several studies from different localities, with estimations ranging from 8.8% up to 29.3% [6,12,13].

Although IGD is rapidly becoming a worldwide public health concern, the literature on this topic remains scant in the context of Saudi Arabia. One study has determined the prevalence of IGD among male adolescents in the Eastern Region [13], but no studies have determined its prevalence among female adolescents. Moreover, no studies have measured this prevalence among adolescents in Al-Ahsa, the largest governorate in the Eastern Region.

Therefore, this study estimates the prevalence and potential risk factors of IGD among female adolescents in Al-Ahsa. It aims to extend the literature on IGD and provide useful recommendations to the concerned authorities.

## Materials and methods

This analytical cross-sectional study was conducted among female secondary school students in Al-Ahsa, Saudi Arabia, between January and February 2023. We estimated the sample size with the Raosoft sample size calculator [14] as 379 using a confidence level of 95%, a margin of error of 5%, and an assumed prevalence of 50% to represent the target population of 23,224 adolescents studying at all-female secondary schools in Al-Ahsa in 2023. We rounded off the estimated sample size to 400.

We used stratified multistage cluster sampling with a probability proportional to size (PPS). First, we distributed the sample size among the four educational sectors based on the number of female secondary school students in each sector in this manner: 89 students from the middle sector (22.1% of the sample size), 98 students from the southern sector (24.6% of the sample size), 127 students from the northern sector (31.8% of the sample size), and 86 students from the eastern sector (21.5% of the sample size). Second, a simple random sample of 20 secondary schools was selected, with five schools selected from each sector. We arbitrarily selected one-fifth of the predetermined number of participants. The sample size of each sector was then distributed among the randomly selected schools according to the total number of students using the PPS technique. Finally, the assigned sample size for each school was distributed among the three grades.

An official letter was sent to the Directorate of Education in Al-Ahsa to facilitate data collection. Then, the principal investigator contacted the coordinators at the selected schools to distribute consent forms to the parents of the students. Next, a self-administered questionnaire (in Arabic) was distributed to the students in electronic or paper format to collect the required data. We also obtained consent from the authors of a previous study conducted in Dammam [13] to use their validated questionnaire. This questionnaire comprised five sections, including items on [a] sociodemographic characteristics (e.g., nationality, age, education, marital status, school performance, etc.); [b] gaming-related behavior (e.g., hours spent playing, monthly expenditure, gaming partner, etc.); [c] the IGDS9‑SF, one of the best robust and concise psychometric tools used to assess individuals with IGD [15]; [d] the Patient Health Questionnaire-9 (PHQ-9), which is a short screening tool for assessing the severity of depression [16]; and [e] the Generalized Anxiety Disorder-7 (GAD-7), which is another short screening tool used to measure the severity of anxiety [17].

We used the same cutoff point as those used in the study conducted in Dammam and others conducted in Korea [13,18]. These studies considered the endorsement of a criterion when "the participant’s response was at the midpoint of the five-point scale: three (sometimes), four (often), or five (very often)" [13,18,19]. Based on this, we classified the status of IGD into three categories. Those who endorsed five or more of the nine criteria in the IGDS9‑SF and received a score between 20 and 45 were considered to have IGD. Those who endorsed three or four of the nine criteria and got a score between 15 and 19 were considered to possibly have IGD. Finally, those who endorsed less than three criteria and got a score of less than 15 were considered regular gamers [13,18]. The variables for anxiety were created based on the score on the GAD-7. Those who scored 1-4, 5-9, 10-14, and 15-21 were considered to have minimal, mild, moderate, and severe anxiety, respectively. The variables for depression were created based on the score on the PHQ-9. Those who scored 1-4, 5-9, 10-14, and 15 ≤ were considered to have minimal, mild, moderate, and severe depression, respectively.

Data entry and analysis were performed using Microsoft Excel and IBM Corp.'s Statistical Package for the Social Sciences (SPSS) v23.0. Categorical variables were presented as frequencies and percentages. Median and interquartile ranges were used to describe scores of anxiety and depression. The chi-squared test was used to compare categorical variables. Pearson correlation coefficients were used to detect associations between IGD, depression, and anxiety scores. Statistical significance was set at 0.05.

Ethical approval (No. 55-EP-2022) was obtained from the Institutional Review Board of King Fahad Hospital, Hofuf, Saudi Arabia.

## Results

The sample comprised 400 female adolescent students. They were divided into two groups based on their answer to the question, "Have you played any video games in the past 12 months?" Most participants (282) answered "yes," while the remaining answered "no." Those who answered "yes" were included in the analysis. Most participants were Saudi nationals (94%) and single (98%). The mean age was 16.91 years (standard deviation [SD] = 1.157 years). Most participants (43%) were studying in the tenth grade, followed by those studying in the twelfth (31%) and eleventh (26%) grades. Regarding school performance, 79% of the students had excellent scores, 18% had very good scores, 4% had good scores, and only 1% had acceptable scores (based on the grading system of the Saudi Ministry of Education: poor ˂ 50%; accepted 50% to ˂ 60%; good, 60% to ˂ 75; very good 75% to ˂ 90%; and excellent ≥ 90%). Approximately 94% of the students did not repeat the grade. Thirty-three percent were absent at least once a week. Regarding participants’ monthly family income, most (37%) did not know it; 23% had an income of less than SAR 5000, 22% had an income of more than SAR 10,000, and 18% had an income between SAR 5000 and SAR 10,000. The fathers of most participants (67%) had completed high school or possessed higher educational qualifications, and most fathers (93%) were employed. The mothers of most participants (74%) had also completed high school or achieved higher educational qualifications, and most mothers (67%) were housewives. Furthermore, there were statistically significant differences among the three groups of gamers based on four sociodemographic characteristics: school performance grade, family’s monthly income, father’s occupation status, and mother’s education level, as shown in Table 1.

**Table 1:** Participants’ sociodemographic characteristics based on the three groups of gamers IGD: Internet gaming disorder; P-IGD: possible Internet gaming disorder; SAR: Saudi Riyal; *: significant

	All gamers (N = 282)						
	Regular (N = 137)	With P-IGD (N = 69)	With IGD (N = 76)	Overall (%)	Chi-square	Degree of freedom	p-value
Nationality							
Saudi, N (%)	127(48.1%)	66(25%)	71(26.9%)	264(100%)	0.676	2	0.71
Non-Saudi, N (%)	10(55.6%)	3(16.7%)	5(27.8%)	18(100%)			
Age (years)							
Mean	-----	------	------	16.91			0.563
Median	-----	------	-------	16			
Standard deviation	------	-------	--------	1.157			
Year of education							
10th, N (%)	54(44.6%)	28(23.1%)	39(32.3%	121(100%)	3.232	4	0.52
11th, N (%)	37(50%)	20(27%)	17(23%)	74(100%)			
12th, N (%)	46(52.9%)	21(24.1%)	20(23%)	87(100%)			
Marital status							
Single, N (%)	135(48.7%)	68(24.5%)	74(26.7%)	277(100%)	0.440	2	0.802
Married, N (%)	2(40%)	1(20%)	2(40%)	5(100%)			
School performance grade							
Excellent, N (%)	117(52.7%)	54(24.3%)	51(23%)	222(100%)	21.207	6	0.002*
Very good, N (%)	18(38.3%)	14(29.8%)	15(31.9%)	47(100%)			
Good, N (%)	2(18.2%)	1(9.1%)	8(72.7%)	11(100%)			
Acceptable, N (%)	0(100%)	0(0%)	2(100%)	2(100%)			
Poor, N (%)	0(100%)	0(0%)	0(0%)	0(0%)			
Grade repetition							
Yes, N (%)	8(44.4%)	4(22.2%)	6(33.3%)	18(100%)	0.398	2	0.82
No, N (%)	129(48.9%)	65(24.6%)	70(26.5%)	264(100%)			
Average absences days							
Attends regularly, N (%)	99(52.4%)	44(23.3%)	46(24.3%)	189(100%)	3.484	2	0.175
Once/week or higher, N (%)	38(40.9%)	25(26.9%)	30(32.2%)	93(100%)			
Father’s education level							
Less than high school, N (%)	25(36.3%)	19(27.5%)	25(36.2%)	69(100%)	9.086	4	0.06
High school/diploma	46(48.4%)	25(26.3%)	24(25.3%)	95(100%)			
University, N (%)	55(59.1%)	19(20.4%)	19(20.4%)	93(100%)			
Family’s monthly income (SAR)							
< 5000 (SAR), N (%)	21(32.8%)	22(34.4%)	21(32.8%)	64(100%)	13.153	6	0.041*
≤ 10,000 (SAR), N (%)	24(54.9%)	10(19.6%)	13(25.5%)	51(100%)			
> 10,000 (SAR), N (%)	39(61.9%)	9(14.3%)	15(23.8%)	63(100%)			
Do not know	49(47.1%)	28(26.9%)	27(26%)	104(100%)			
Mother’s occupation status							
Working, N (%)	39(44.8%)	27(31%)	21(24.1%)	86(100%)	2.392	2	0.302
Housewife, N (%)	95(50.5%)	42(22.3%)	51(27.1%)	188(100%)			
Father’s occupation status							
Employed, N (%)	126(52.3%)	55(22.8%)	60(24.9%)	241(100%)	11.951	2	0.003*
Unemployed, N (%)	3(14.3%)	7(33.3%)	11(52.4%)	21(100%)			
Mother’s education level							
Less than high school, N (%)	22(32.8%)	18(26.9%)	27(40.3%)	67100%)	16.452	4	0.002*
High school/diploma, N (%)	57(60%)	16(16.8%)	22(23.2%)	95(100%)			
University, N (%)	47(46.5%)	32(31.7%)	22(21.8%)	101(100%)			

Eighty-eight percent of the participants played for four hours or less daily on weekdays, while 76% played for four hours or less daily on weekends. Most students (97%) spent less than Saudi Riyal (SAR) 200 per month on video games. Approximately 71% of the students started playing video games between the ages of seven and 15. We found a statistically significant difference in the gaming behavior of the three groups of gamers, except for their behavior concerning a gaming partner and money expenditure on video games. Most participants (72%) played online games. Based on the definition that those who endorse five or more out of the nine criteria by answering "sometimes," "often," or "very often" should be considered as having IGD, the prevalence of IGD among female high school students in Al-Ahsa was estimated to be 19%. If those with possible IDG were also included, the prevalence would have reached 36.25%, as shown in Table 2.

**Table 2:** Participants’ gaming behavior based on the three groups of gamers IGD: Internet gaming disorder; P-IGD: possible Internet gaming disorder; SAR: Saudi Riyal; *: significant

	Regular (N = 137)	With P-IGD (N = 69)	With IGD (N = 76)	Overall (%)	Chi-square test	Degree of freedom	p-value
Hours spent playing videogames per day on weekdays							
4 hours or less, N (%)	131(52.6%)	61(24.5%)	57(22.9%)	249(100%)	20.117	2	0.001*
> 4 hours, N (%)	6(18.2%)	8(24.2%)	19(57.6%)	33(100%)			
Hours spent playing videogames per day on weekends							
4 hours or less, N (%)	119(55.9%)	49(23%)	45(21.1%)	213(100%)	21.231	2	0.001*
> 4 hours, N (%)	18(26.1%)	20(29%)	31(44.9%)	69(100%)			
Monthly expenditure on video games (Saudi Riyal)							
Less than SAR 200, N (%)	135(49.3%)	67(24.5%)	72(26.3%)	274(100%)	2.567	2	0.277
More than SAR 200, N (%)	2(25%)	2(25%)	4(50%)	8(100%)			
Age at which one started playing electronic games (years)							
< 7, N (%)	9(27%)	16(49%)	8(24%)	33(100%)	13.281	6	0.039*
7–10, N (%)	57(53%)	20(18%)	31(29%)	108(100%)			
11–15, N (%)	47(51%)	21(23%)	24(26%)	92(100%)			
> 15, N (%)	24(49%)	12(24%)	13(27%)	49(100%)			
Gaming partner							
Play alone, N (%)	62(47.7%)	30(23.1%)	38(29.2%)	130(100%)	0.695	2	0.706
Play with a partner, N (%)	75(49.3%)	39(25.7%)	38(25%)	152(100%)			
Connectivity to the Internet							
Online, N (%)	98(44.3%)	55(24.9%)	68(30.8%)	221(100%)	9.378	2	0.01*
Offline, N (%)	39(63.9%)	14(23%)	8(13.1%)	61(100%)			

Based on participants’ scores on the PHQ-9, 63.2% of participants with IGD, 33% of participants with possible IGD, and 17.5% of regular gamers had moderate to severe depression. Based on participants’ scores on the GAD-7, approximately 47.4% of participants with IGD, 30.4% of participants with possible IGD, and 11.7% of regular gamers had moderate to severe anxiety. Depression and anxiety scores were statistically and significantly different between the three groups of gamers, as shown in Table 3.

**Table 3:** Participants’ depression and anxiety scores based on the three groups of gamers IGD: Internet gaming disorder; P-IGD: possible Internet gaming disorder; IQR: interquartile range; PHQ‑9: Patient Health Questionnaire‑9; GAD‑7: Generalized Anxiety Disorder‑7; *: significant

	All gamers (N = 282)					
	Regular (N = 137)	With P-IGD (N = 69)	With IGD (N = 76)	Chi-square	Degree of freedom	p-value
Depression (PHQ-9)						
Median (IQR)	7 (9)					
No/minimal, N (%)	78 (56.9%)	17 (24.6%)	8 (10.5%)	117.114	48	< 0.001*
Mild, N (%)	35 (25.5%)	29 (42%)	20 (26.3%)			
Moderate, N (%)	15 (10.9%)	17 (24.6%)	23 (30.3%)			
Severe, N (%)	9 (6.6%)	6 (8.7%)	25 (32.9%)			
Anxiety (GAD-7)						
Median (IQR)	5 (8)					
No, N (%)	95 (69.3%)	29 (42%)	11 (14.5%)	118.370	42	< 0.001*
Mild, N (%)	26 (19%)	19 (27.5%)	29 (38.2%)			
Moderate, N (%)	9 (6.6%)	17 (24.6%)	18 (23.7%)			
Severe, N (%)	7 (5.1%)	4 (5.8%)	18 (23.7%)			

The mother’s education level above high school (adjusted odds ratio (OR) = 0.65 (confidence interval (CI): 0.443-0.955), p-value = 0.028), family’s monthly income exceeding SAR 10000 (adjusted OR = 0.49 (CI: 0.261-0.919), p-value = 0.026), employed father (adjusted OR = 0.160 (CI: 0.045-0.565), p-value = 0.004), spending more than four hours/day playing video games on weekdays (adjusted OR = 4.788 (CI: 1.893-12.109), p-value < 0.001), spending more than four hours/day playing video games on weekends (adjusted OR = 3.531 (CI: 1.911-6.525), p-value < 0.001), began playing video games at less than seven years of age (adjusted OR = 2.781 (CI: 1.213-6.372), p-value = 0.016), employed mother (adjusted OR = 2.467 (CI: 1.233-4.933), p-value = 0.011), depression (adjusted OR = 4.113 (CI: 2.270-7.451), p-value < 0.001), anxiety (adjusted OR = 4.735 (CI: 2.413-9.290), p-value < 0.001), and playing an online game (adjusted OR = 2.186 (CI: 1.210-3.950), p-value = 0.010) were found to be statistically and significantly associated with IGD. It was also found that five of them have the potential to increase the risk of one developing IGD by more than two times: spending more than four hours/ day playing video games, beginning to play video games at less than seven years of age, playing an online game, and having depression or anxiety. On the other hand, some factors can be protective factors, such as the parent's education level being higher than the high school level and the monthly family income being more than SAR 10,000 (Table 4).

**Table 4:** Logistic regression analysis: final model correlates of internet gaming disorder Ref: the covariate reference category; SAR: Saudi Riyal; *: significant

Covariate	N	Odds ratio	95% Confidence interval	p-value
Father’s education level				
Less than high school	69	0.759	0.433-1.33	0.337
Above high school	188	(Ref)	(Ref)	(Ref)
Mother’s education level				
Less than high school	67	0.65	0.443-0.955	0.028*
Above high school	196	(Ref)	(Ref)	(Ref)
Family’s monthly income				
Less than SAR 10000	219	0.49	0.261-0.919	0.026*
Exceeding SAR 10000	63	(Ref)	(Ref)	(Ref)
Hours spent playing videogames per day on weekdays				
More than 4 hours/day	33	(Ref)	(Ref)	(Ref)
Less than 4 hours/day	249	4.788	1.893-12.109	< 0.001*
Hours spent playing videogames per day on weekends				
More than 4 hours/day	69	(Ref)	(Ref)	(Ref)
Less than 4 hours/day	213	3.531	1.911-6.525	< 0.001*
Began playing video games at				
Less than seven years of age	33	(Ref)	(Ref)	(Ref)
More than seven years of age	249	2.781	1.213-6.372	0.016*
Mother’s occupation status				
Employed	87	(Ref)	(Ref)	(Ref)
Unemployed	188	2.467	1.233-4.933	0.011*
Father’s education level				
Unemployed	21	0.160	0.045-0.565	0.004*
Employed	261	(Ref)	(Ref)	(Ref)
Depression				
No/minimal-Mild/moderate	187	4.113	2.270-7.451	< 0.001*
Moderately severe/severe	95	(Ref)	(Ref)	(Ref)
Anxiety				
No, Mild	209	4.735	2.413-9.290	< 0.001*
Moderate, Severe	73	(Ref)	(Ref)	(Ref)
Playing an online game				
Online	221	(Ref)	(Ref)	(Ref)
Offline	61	2.186	1.210-3.950	< 0.001*

**Figure 1:**
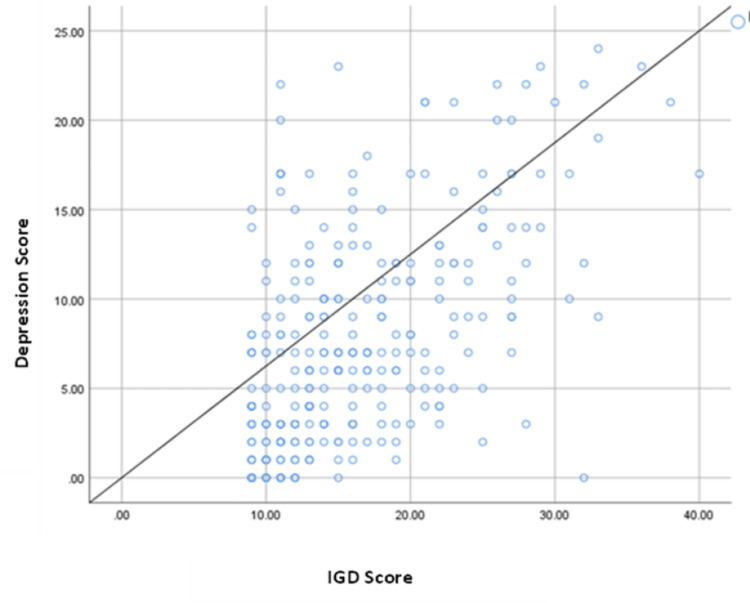
Correlation between participants’ IGD and depression scores (Pearson correlation coefficient = 0.549, p-value ≤ 0.001) IGD: Internet gaming disorder

The correlation between IGD and depression scores was statistically significant, with a moderately positive Pearson correlation coefficient (0.549, p-value ≤ 0.001), as shown in Figure 1.

**Figure 2:**
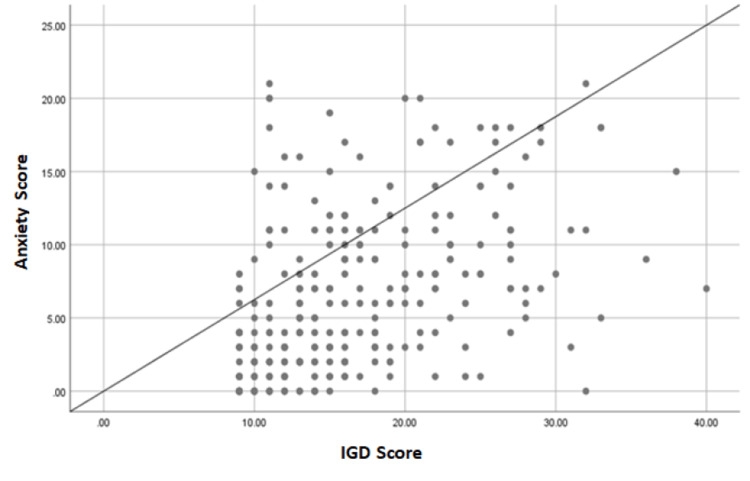
Correlation between participants’ IGD and anxiety scores (Pearson correlation coefficient = 0.461, p-value ≤ 0.001) IGD: Internet gaming disorder

There was a statistically significant and moderately positive correlation between IGD and anxiety scores (0.461, p-value = 0.001) as shown in Figure 2.

**Figure 3:**
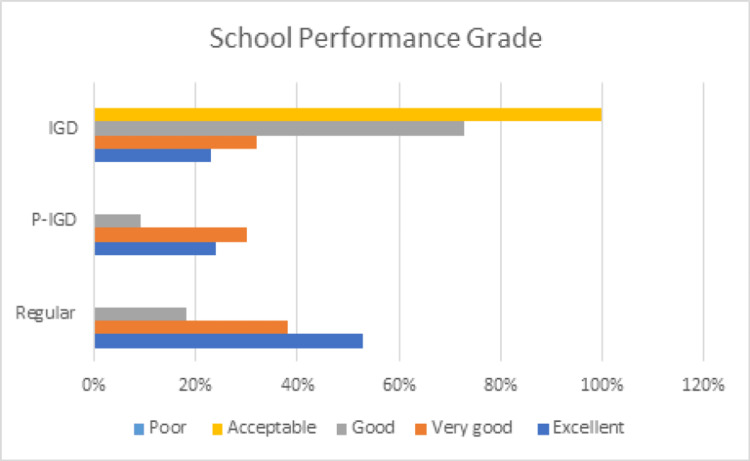
School performance grade among the three groups of gamers IGD: Internet gaming disorder; P-IGD: possible Internet gaming disorder

There were differences in the school performance grade among the three groups of gamers, with the regular gamer having the highest percentage of students who got an excellent grade (53%), while the lowest percentage was in the IGD group (23%), as shown in Figure 3.

## Discussion

This study measured the prevalence of IGD among female secondary school students in Al-Ahsa based on the criteria the APA proposes. It also determined the potential risk factors for IGD. The results show that the prevalence rate of IGD is 19%, which means that approximately one in every five female secondary school students in Al-Ahsa has IGD. Most studies on IGD in Asia have been conducted in Singapore, South Korea, and China [20]. Recently, a study measured the prevalence of IGD in three Arab countries, Jordan, Syria, and Kuwait, and found that the prevalence of IGD is 5.3%, 6.1%, and 7.8%, respectively [21]. However, such data remains limited in the context of Saudi Arabia. A review of the literature on the prevalence of IGD in Saudi Arabia yielded several studies. The first study was conducted among 228 medical students at King Saud University, Riyadh. It reported a prevalence rate of 8.8%, with 19.3% of the participants being risky gamers. No statistically significant association was found between IGD and age, sleep pattern, or academic achievement [12]. The second study was conducted among 450 adolescent students from intermediate and secondary schools in the Faifa Governorate in the southern region of Saudi Arabia. The researchers found a 29.3% prevalence of IGD [6]. The third study was conducted among 798 male students at secondary schools in Dammam, and it reported a 21.85% prevalence of IGD [13]. Another study that estimated the prevalence of IGD in randomly selected Saudi Arabian universities found the prevalence to be 10.1% [22]. The differences in the prevalence of IGD across these studies can be attributed to differences in the demographic characteristics of the study population, the study area, and the assessment tools [9].

The results of executing the final logistic regression model revealed that some factors can increase the risk of developing IGD, such as playing video games for prolonged periods [20, 23], particularly for more than four hours daily, beginning to play video games at an early age [24], having depression [25-27], having anxiety [26], and playing online games [27]. Conversely, some factors can reduce the likelihood of one developing IGD, such as the parents’ education level being higher than the high school level and the monthly family income being more than SAR 10,000.

We also found a statistically significant association between IGD scores and depression and anxiety scores. Many studies have found this association [13,18,26]. However, the direction of the association needs further evaluation, as it could be considered a risk factor or consequence of IGD [20]. IGD is considered the most prominent subtype of internet addiction [28]. One study suggested that those who are diagnosed with generalized anxiety disorder are prone to developing addictive behaviors as a coping mechanism [28]. Some studies have shown an association between Internet addiction and mental disorders such as anxiety and depression [28]. Furthermore, several studies have revealed that IGD is associated with depression and anxiety. This association can stem from the vulnerability of those with mental disorders, as they may develop addictive behaviors as a coping mechanism.

Our results showed that significant differences exist in the school performance of the three groups of gamers. Therefore, school performance should be considered a consequence of IGD [20,29]. The student group with IGD had the highest percentage of students with good grades but the lowest percentage of students with excellent grades. This result can stem from the criteria for diagnosing IGD, such as dependency on video games and time spent playing video games [30].

This study is not free of limitations. First, it has a cross-sectional design, which is not the best design to identify the factors associated with IGD. Second, the sample was small, and a larger sample size would be required to increase the accuracy of our results. Also, although we used a random method of sampling, the enrolled participants were not homogeneous regarding certain characteristics, such as their school performance, family’s monthly income, father’s occupation status, and mother’s education level. The inclusion of only adolescent females could also limit the generalizability of the results to the whole population. Despite these limitations, this study is the first to investigate IGD in the eastern region of Saudi Arabia, particularly among female high school students. Moreover, most of its findings have been supported by studies worldwide.

## Conclusions

Internet gaming disorder is a significant health problem among female students in Al-Ahsa, Saudi Arabia. Multiple factors increase the likelihood of developing IGD, and they include playing video games for more than four hours daily, beginning to play video games at an early age, having depression or anxiety, and playing online games. Factors that can reduce the likelihood of developing IGD include the high parents’ education level and the elevated monthly family income.

We recommend implementing a well-prepared awareness project for primary to secondary school students as well as involving the parents because of the significant role they have in shaping their children’s internet gaming behavior. We emphasize the importance of teaching children behavioral strategies that strengthen their control over their actions. Finally, we recommend conducting an observational study to determine the direction of the relationship between IGD and mental and social health problems, such as depression and anxiety.
